# Natural polymorphisms in *ZmIRX15A* affect water‐use efficiency by modulating stomatal density in maize

**DOI:** 10.1111/pbi.14153

**Published:** 2023-08-12

**Authors:** Kun Zhang, Ming Xue, Feng Qin, Yan He, Yuyi Zhou

**Affiliations:** ^1^ State Key Laboratory of Plant Physiology and Biochemistry Engineering Research Center of Plant Growth Regulator College of Agronomy and Biotechnology China Agricultural University Beijing China; ^2^ Jiangsu Key Laboratory of Crop Genetics and Physiology Co‐Innovation Center for Modern Production Technology of Grain Crops Key Laboratory of Plant Functional Genomics of the Ministry of Education Yangzhou University Yangzhou China; ^3^ State Key Laboratory of Plant Physiology and Biochemistry College of Biological Sciences China Agricultural University Beijing China; ^4^ National Maize Improvement Center of China College of Agronomy and Biotechnology China Agricultural University Beijing China

**Keywords:** Stomata, water‐use efficiency, *ZmIRX15A*, genome‐wide association, maize

## Abstract

Stomatal density (SD) is closely related to crop drought resistance. Understanding the genetic basis for natural variation in SD may facilitate efforts to improve water‐use efficiency. Here, we report a genome‐wide association study for SD in maize seedlings, which identified 18 genetic variants that could be resolved to seven candidate genes. A 3‐bp insertion variant (InDel1089) in the last exon of *Zea mays* (*Zm*) *IRX15A* (*Irregular xylem 15A*) had the most significant association with SD and modulated the translation of *ZmIRX15A* mRNA by affecting its secondary structure. Dysfunction of *ZmIRX15A* increased SD, leading to an increase in the transpiration rate and CO_2_ assimilation efficiency. *ZmIRX15A* encodes a xylan deposition enzyme and its disruption significantly decreased xylan abundance in secondary cell wall composition. Transcriptome analysis revealed a substantial alteration of the expression of genes involved in stomatal complex morphogenesis and drought response in the loss‐of‐function of *ZmIRX15A* mutant. Overall, our study provides important genetic insights into the natural variation of leaf SD in maize, and the identified loci or genes can serve as direct targets for enhancing drought resistance in molecular‐assisted maize breeding.

## Introduction

Maize (*Zea mays* L.) is one of the most important crops worldwide due to its high potential for providing food, forage, and industrial products. However, maize production is frequently compromised by water scarcity as a result of climate warming and erratic rainfall patterns on a global scale (Lobell *et al*., 2014). The amount of CO_2_ fixed by photosynthesis relative to the amount of water vapour lost to the atmosphere is termed water‐use efficiency (WUE). Stomata are adjustable pores on the leaf surface that regulate WUE through their regulation of CO_2_ uptake and water loss (Hetherington and Woodward, 2003). Recent studies have demonstrated the feasibility of improving WUE—and hence drought tolerance—by reducing stomatal density (SD) on leaves of several plant species including *Arabidopsis* (Guo *et al*., 2019; Wang *et al*., 2021; Yoo *et al*., 2010), tobacco (*Nicotiana tabacum*; Yu *et al*., 2008), *Solanum lycopersicum* (Li *et al*., 2020), poplar (Wang *et al*., 2016a), maize (Xiang *et al*., 2021) and wheat (Dunn *et al*., 2019). Thus, understanding the molecular mechanisms underlying stomata formation and identifying functional variations that underlie their morphological characteristics (SD phenotyped in this research) are critical for improving maize WUE.

Stomata formation begins with protodermal cells that differentiate sequentially to produce meristemoid mother cells, meristemoid cells, guard mother cells, and guard cells, thus ultimately forming functional guard cells (Chater *et al*., 2017; Pillitteri *et al*., 2007; Zoulias *et al*., 2018). This developmental progression is regulated by many factors such as SDD1, TMM, SPCH, MUTE, and FAMA (Dow and Bergmann, 2014; McKown and Bergmann, 2020; Woolfenden *et al*., 2018). In contrast to the dicotyledonous *Arabidopsis*, the stomata of monocots such as maize, rice, barley and *Brachypodium* contain two dumbbell‐shaped guard cells flanked by two subsidiary cells, which develop in parallel rows within defined epidermal cell lines in a base‐to‐tip orientation (Chen *et al*., 2017). Using a reverse genetics approach, the functions of orthologs of *Arabidopsis* SPCH, MUTE, FAMA, ICE and SCRM2 have been characterized in several grass species, manifesting a conserved stomatal regulatory mechanism between cereal grasses and *Arabidopsis* (Liu *et al*., 2009; McKown and Bergmann, 2020; Raissig *et al*., 2017; Wu *et al*., 2019). In addition, many recent studies have shown that cell wall‐related genes play an important role in dumbbell‐shaped morphogenesis of developing and mature stomata (Amsbury *et al*., 2016; Chen *et al*., 2021; Sun *et al*., 2022a). During stomata development, the cell wall undergoes dynamic changes, such as the formation of a new cell wall during the symmetrical division of guard mother cells, the degradation of the cell wall between two adjacent guard cells to form pores and thickening of the guard cell wall at stomatal maturation (Apostolakos, 2004; Rui *et al*., 2018; Spiegelhalder and Raissig, 2021).

To thrive in a wide variety of ecosystems on an evolutionary timeframe, stomatal characteristics have undergone extensive natural variation among different plant subgroups (Drake *et al*., 2013; Taylor *et al*., 2012). In this regard, stomatal number and size vary substantially among different *Oryza* species and *Oryza* stomatal complex. For example, the *Oryza sativa* complex exhibits the greatest diversity in SD, whereas the *O. officinalis* complex has greater diversity in stomatal size (Chatterjee *et al*., 2020). In addition, a study has used automated confocal microscopy methods elucidating the substantial genetic variation that underlies both stomata size and density in *Arabidopsis* (Dittberner *et al*., 2018). Moreover, the genetic basis for the SD variation in sorghum was recently reported (Bheemanahalli *et al*., 2021; Ferguson *et al*., 2021). However, little is known about the genetic basis for natural variation of SD in maize, knowledge of which could help breeders improve drought resistance.

Here, we report a genome‐wide association study (GWAS) of maize SD at the seedling stage in a maize association panel including 424 inbred lines with tropical, subtropical, and temperate backgrounds (Yang *et al*., 2011). The most significant variation was found to occur within the gene, namely *Zea mays IRX15A* (*ZmIRX15A*), which is putatively involved in xylan biosynthesis in the plant cell wall (Brown *et al*., 2009, 2011; Jensen *et al*., 2014; Wu *et al*., 2009). A 3‐bp insertion variant (InDel1089; InDel, insertion and deletion) in the last exon of *ZmIRX15A* was significantly associated with SD. The InDel1089 polymorphism influenced the translation of *ZmIRX15A* mRNA by affecting its secondary structure. In addition, *ZmIRX15A* negatively regulated WUE and drought tolerance by altering the SD. Transcriptome analysis also revealed that loss‐of‐function of *ZmIRX15A* resulted in the differential expression of genes responsible for stomatal complex morphogenesis and drought response. These findings provide an in‐depth understanding of the natural variation in SD and reveal an important genetic target for enhancing drought resistance during maize breeding.

## Results

### GWAS for maize stomatal density at the seedling stage

**Figure 1:**
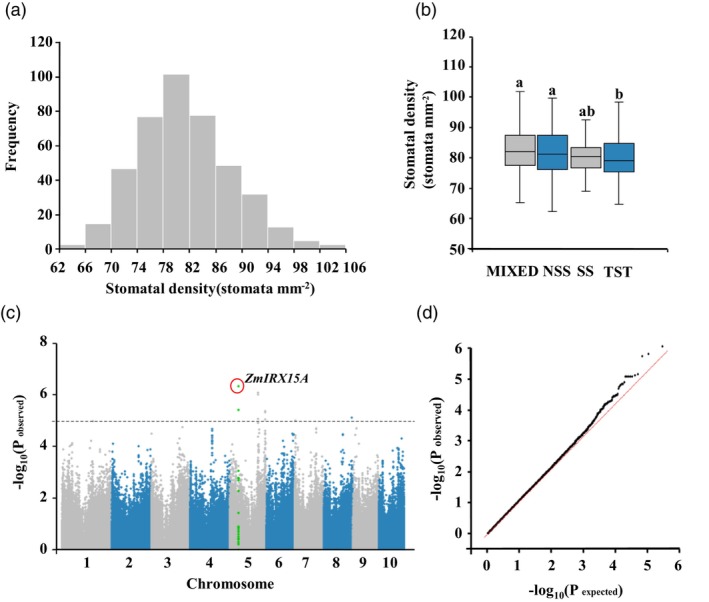
GWAS for maize stomatal density and size at the seedling stage. (a) Frequency distribution of SD. Classes of trait values are shown on the *x*‐axis, and counts of inbred lines with the phenotypic values for these bins are shown on the *y*‐axis. (b) Boxplot of SD distribution in different subpopulations. The Kruskal–Wallis test was applied to examine trait differences among subpopulations. Different letters indicate significance levels at *P* ≤ 0.05. The number of inbred lines included in each subpopulation are 180, 54, 27 and 163 for the MIXED, NSS, SS and TST, respectively. (c) Manhattan plot for a genome‐wide association study (GWAS) of SD from the maize association‐mapping panel. The dashed horizontal line depicts the Bonferroni‐adjusted significance threshold (*P* = 1.0 × 10^−5^). (d) Quantile–quantile plot for the GWAS under a mixed linear model (MLM).

To identify genes associated with SD, we performed a GWAS by analysing a natural maize association population that included 163 tropical/subtropical (TST) inbred lines, 81 temperate lines (TEM, including SS and NSS), and 180 lines of mixed origin (MIXED; Li *et al*., 2013; Yang *et al*., 2011). SD was found to vary substantially, ranging from 62.44 to 101.89 mm^−2^ (Figure 1A). The mean value of SD in the TST subpopulation was much less than that in the MIXED and TEM subpopulations, suggesting that maize‐inbred lines of TST origin tend to have lower SD (Figure 1B; Table S1). Approximately 560 000 single nucleotide polymorphisms (SNPs) with a minor allele frequency of ≥0.05 were applied for further analysis (Li *et al*., 2013; Yang *et al*., 2011). Under the standard mixed linear model, which accounts for false positives arising from the population structure and kinship of the natural variation in the population, 18 SNPs were significantly associated with SD (Figure 1C,D; Table S2). The most significant SNP, namely chr5.S_50450734 (in the last exon of *Zm00001d014532*), accounted for ~7% of the phenotypic variance, whereas other individual loci contributed at most ~5% of the variation of the entire population.

### *ZmIRX15A* is significantly associated with stomatal density

**Figure 2:**
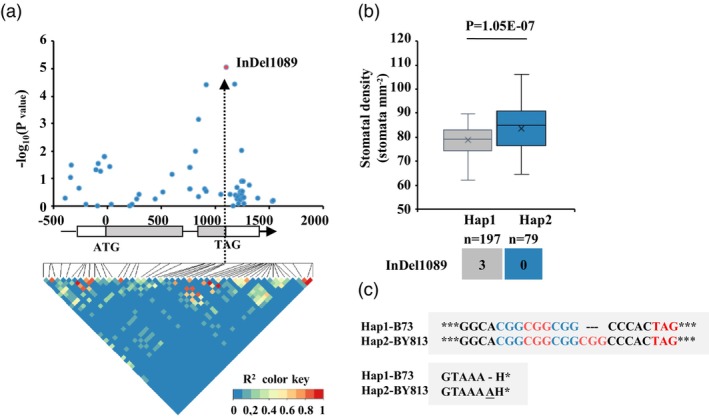
*ZmIRX15A* is significantly associated with stomatal density. (a) The association analysis of genetic variation in *ZmIRX15A* with SD in maize and the pattern of pairwise linkage disequilibrium of DNA polymorphisms. The most significant Indel (InDel1089) in the exon is highlighted in red and connected to its location in the gene diagram by dotted lines. (b) The SD distribution of each haplotype group is displayed by the box plot. Haplotypes of *ZmIRX15A* among maize natural variants. *n*: denotes the number of genotypes belonging to each haplotype group. Statistical significance was determined by a two‐sided *t*‐test. (c) Schematic diagram of different haplotype DNA and amino‐acid sequences proximal to InDel1089.

A BLASTP search of the NCBI database revealed that the protein encoded by *Zm00001d014532* belongs to the family of enzymes involved in polysaccharide biosynthesis (*Posacc_synt_4*, Figure S1), sharing 51% amino‐acid sequence identity with the xylan deposition enzyme *AtIRX15* of *Arabidopsis* (Brown *et al*., 2011; Jensen *et al*., 2011). Thus, we designated *Zm00001d014532* as *ZmIRX15A*. To fully characterize genetic variations in *ZmIRX15A*, we resequenced alleles of *ZmIRX15A* from 275 maize‐inbred lines, including 350 bp upstream of the 5′ untranslated region (UTR), the 252‐bp 5′UTR, the 1094‐bp coding sequence, the 428‐bp 3′UTR, and the 100 bp downstream of the 3′UTR. An additional 40 SNPs and 14 InDels were discovered (minor allele frequency ≥0.05; Table S3). To investigate whether natural variations in *ZmIRX15A* contribute to the variation of SD in maize, we performed an association analysis for all these SNPs/InDels with SD phenotypes using TASSEL (Bradbury *et al*., 2007; Yu *et al*., 2006). InDel1089 resulting in an insertion of the amino‐acid alanine without any frameshift mutation exhibited the most significant association with maize SD (*P* = 8.50E−06, Figure 2a). Then, the 275 maize genotypes were classified into two haplotype groups based on the statistically significant association of variants with SD (Figure 2b). *ZmIRX15A* ^B73^ is a representative of haplotype group 1 (Hap1, *n* = 197), whereas *ZmIRX15A* ^BY813^ belongs to Hap2 (*n* = 45; Figure 2c). The SD was significantly lower in the Hap1 lines than in the Hap2 lines (Wilcoxon rank‐sum test, *P* = 1.05E−07; Figure 2b). Together, these results suggested that *ZmIRX15A* is significantly associated with SD.

### InDel1089 polymorphism modulates the translation of *ZmIRX15A* mRNA by affecting its secondary structure

To address whether variations in SD resulted from differential *ZmIRX15A* transcription, an expression quantitative trait locus analysis for *ZmIRX15A* was carried out with the same association population using RNA sequencing data (Liu *et al*., 2020). No significant genetic variations within the *ZmIRX15A* region were found to be associated with the mRNA level (Figure S2A). In addition, no other polymorphisms identified by resequencing were significantly associated with *ZmIRX15A* mRNA level (Figure S2B). Consistently, there was no significant difference in *ZmIRX15A* mRNA abundance between Hap1 (*n* = 102) and Hap2 (*n* = 26; Wilcoxon rank‐sum test, *P* = 0.25; Figure S2C). Moreover, quantitative reverse transcription‐PCR (qRT‐PCR) analysis revealed that *ZmIRX15A* mRNA level was comparable between 10 randomly selected inbred lines of Hap1 and those of Hap2 (permutation test, *P* = 0.18; Figure S2D). These findings indicated that natural variation in SD is not resulted from differential transcription of *ZmIRX15A*, but more likely a consequence of post‐transcriptional regulation.

**Figure 3:**
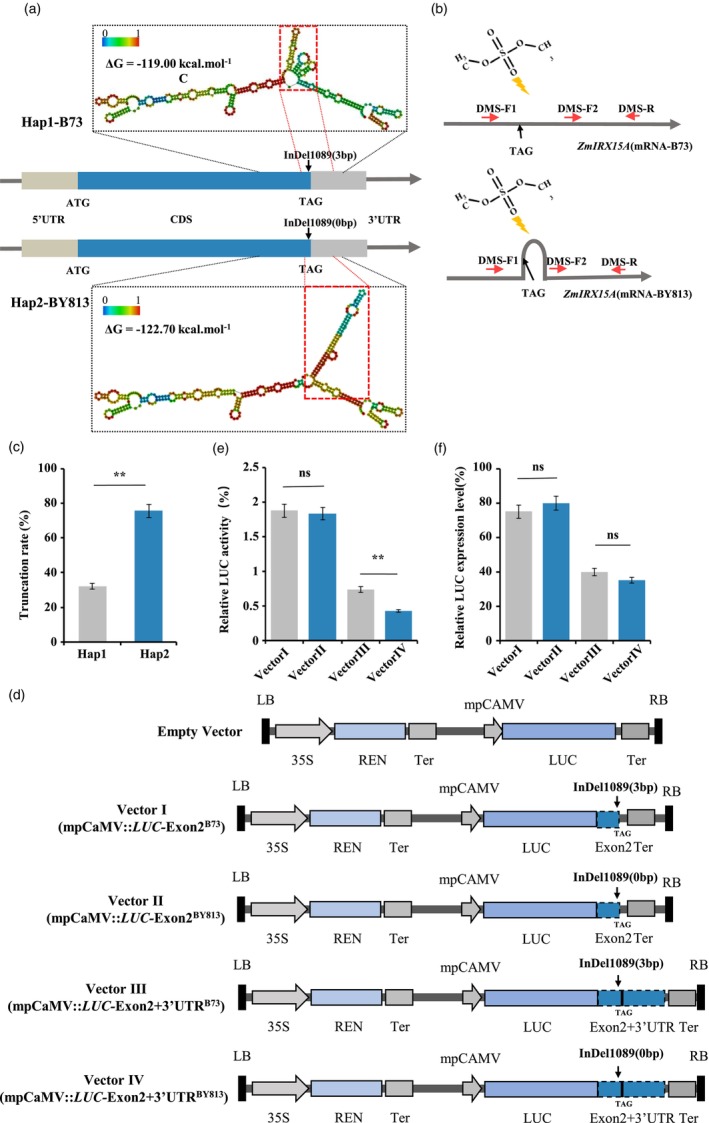
Effect of InDel1089 on *ZmIRX15A* mRNA secondary structure and attribution to differential translation efficiency. (a) Schematic view of the minimum free energy model of the mRNA secondary structures. These structures were predicted by inputting a sequence of 300 bp before and after InDel1089 of *ZmIRX15A* mRNA. The minimum free energy is indicated. The arrow indicates the position of InDel1089. (b) Diagram of the dimethyl sulphate (DMS)–primer extension–quantitative reverse transcription (qRT)‐PCR method for validating the RNA secondary structure in the particular sequence of the *ZmIRX15A* mRNA. Red arrows mark the primers used in this experiment. TAG, stop codon. (c) Primer‐extension assay coupled with qRT‐PCR validates the extent of DMS modifications on Hap1 and Hap2 *ZmIRX15A* mRNAs. (d) Constructs used to test the effect of InDel1089 on *ZmIRX15A* mRNA translation in transient expression assays in maize leaf protoplasts. LB, left border; LUC, firefly luciferase; RB, right border; REN, *Renilla* luciferase; Ter, terminator. (e) Comparison of LUC activity of different vectors. (f) Relative LUC expression level was calculated as the ratio of the LUC and REN expression in each construct. Error bars indicate the SE of the mean. Values represent the mean ± SD (*n* = 3). **P < 0.01; ns, not statistically significant. Student's *t*‐test and Bonferroni correction for multiple tests.

To study whether microRNA binding, mRNA folding, or regulatory RNA elements were altered by InDel1089, we performed a bioinformatics analysis using RegRNA 2.0 and the RNAfold web server (Chang *et al*., 2013; Gruber *et al*., 2008). RegRNA 2.0 analysis of the *ZmIRX15A* mRNA sequence revealed that the polymorphism was neither located within a microRNA binding site nor hampered generation of a putative regulatory RNA element(s) such as AU‐rich motifs. The RNAfold results suggested that InDel1089 markedly affected the *IRX15A* mRNA secondary structure (Figure S2E). *ZmIRX15A* mRNA (Hap2‐BY813) tended to form a lengthy stem‐loop structure around the TAG site instead of three short stem‐loop structures (Hap1‐B73; Figure 3a). In addition, free energy prediction of mRNA secondary structure revealed that the Hap2 *ZmIRX15A* mRNA was more stable than the Hap1 *ZmIRX15A* mRNA (Figure 3a). It has been well known that less RNA secondary structure of minimum free energy (MFE) correlates with higher mRNA stability and translation efficiency (Akdeli *et al*., 2014; Jiang *et al*., 2021; Mauger *et al*., 2019; Wang *et al*., 2006). As a result, *ZmIRX15A* Hap2 mRNA tended to have a higher probability of forming a relatively more stable secondary structure, presumably affecting the translation of *ZmIRX15A* mRNA.

To provide experimental support for the difference in the predicted RNA structure model, treatment with dimethyl sulphate (DMS) coupled with primer extension during reverse transcription was performed *in vivo* (Ding *et al*., 2014; Zhu *et al*., 2021). DMS alkylates the Watson‐Crick face of unpaired adenosine (A) and cytosine (C). Modified A and C residues generally block reverse transcription, resulting in a truncated cDNA. As shown in Figure 3b, DMS treatment led to a decrease in longer cDNAs with an increase in truncations. More importantly, the truncation rate was significantly higher for Hap2 *ZmIRX15A* mRNA relative to Hap1 mRNA (permutation test, *P* < 0.05; Figure 3c), supporting the prediction that the InDel1089 allele indeed altered the secondary structure of *ZmIRX15A* mRNA.

mRNA stability correlates with the cellular abundance of the corresponding protein (Karthi *et al*., 2017). To further investigate the functional relevance of InDel1089 at the level of translation, DNA sequences of *ZmIRX15A* from inbred lines B73 and BY813 were cloned downstream of the luciferase (*LUC*) open‐reading frame under the minimal promoter of the mpCaMV to construct four reporter vectors: mpCaMV‐LUC::Exon2^B73^ (Vector I), mpCaMV‐LUC::Exon2^BY813^ (Vector II), mpCaMV::LUC‐Exon2 + 3′UTR^B73^ (Vector III), and mpCaMV::LUC‐Exon2 + 3′UTR^BY813^ (Vector IV; Figure 3d). Maize mesophyll protoplasts were transiently transfected with each of the recombinant *LUC* vectors, as described previously (Huang *et al*., 2018), and translation was measured as the ratio of LUC to *Renilla* luciferase (REN) activity. LUC activity did not differ significantly between Vectors I and II, although Vector III exhibited almost twice higher LUC activity than Vector IV (*t*‐test, *P* = 6E−04), indicating that the greater LUC activity of Vector III resulted from the mRNA secondary structure variation at the InDel1089 locus (Figure 3e). Notably, comparison of relative LUC expression in these vectors (i.e. Vector I vs. Vector II; Vector III vs. Vector IV) revealed that the InDel1089 polymorphism did not influence LUC expression (Figure 3f). We concluded that InDel1089 could modulate the translation of *ZmIRX15A* mRNA by affecting its mRNA secondary structure, thereby leading to natural variation in SD.

### *ZmIRX15A* dysfunction increases stomatal density by altering cell wall composition

A phylogenetic tree was constructed for *ZmIRX15A* proteins and their orthologs from rice, sorghum, and *Arabidopsis*. We found another *IRX15* ortholog in maize, namely *ZmIRX15B*, which was not on the same branch as *ZmIRX15A* (Figure S3A). Tissue‐specific analysis revealed that *ZmIRX15B* was highly expressed in roots but only at low levels in other organs. In contrast, *ZmIRX15A* was constitutively expressed in all organs except pollen (Figure S3B). These results suggested that *ZmIRX15A* has likely diverged from *ZmIRX15B* in maize. In addition, transient expression of UBQ10:*ZmIRX15A*‐GFP in tobacco leaf cells showed that *ZmIRX15A* localized in the nucleus and plasma membrane (Figure S3C).

**Figure 4:**
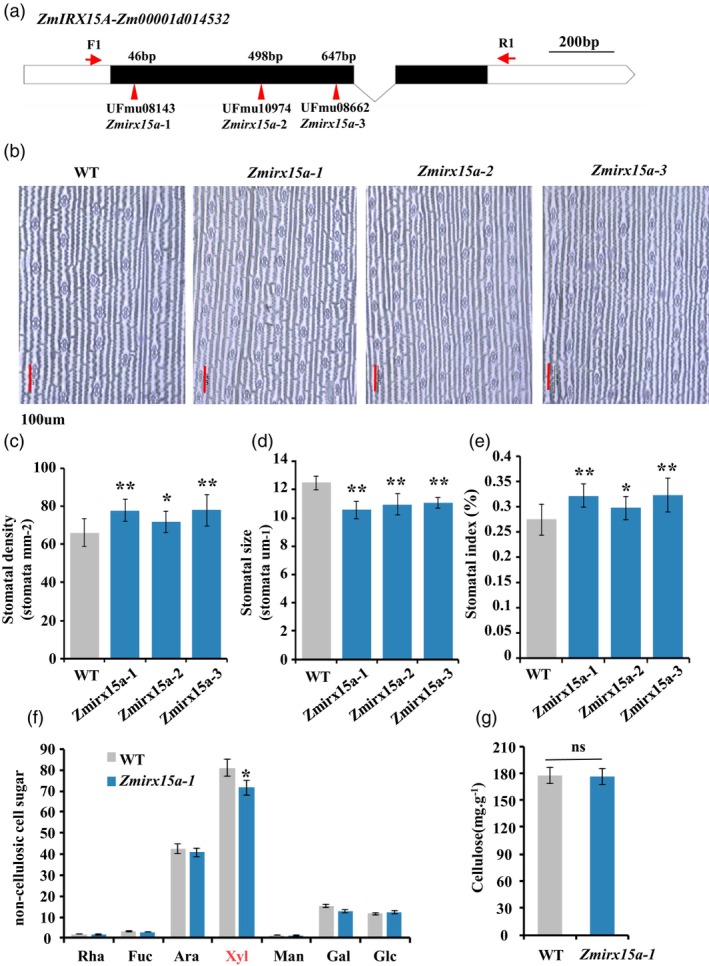
*ZmIRX15A* dysfunction increases stomatal density by altering cell wall composition. (a) Schematic structure and mutation site of *ZmIRX15A*. The lines, black boxes, and white boxes represent introns, coding sequences, and UTR regions, respectively. The red triangle indicates the Mu insertion. Scale bar = 200 bp. (b) Images of the abaxial epidermis of the third leaf of well‐watered plants at the three‐leaf stage for the wild‐type (WT) and *ZmIRX15A*‐mu mutants. Scale bars, 100 μm. (c) Stomatal density (SD). (d) Stomatal size. (e) Stomatal index (SI). (f) Sugar composition of non‐cellulosic cell wall carbohydrates for *Zmirx15‐1* and the corresponding WT parents (*n* = 3). Individual rhamnose (Rha), fucose (Fuc), arabinose (Ara), xylose (Xyl), mannose (Man) and galactose (Gal) are expressed as a percentage of the cell wall fraction. (g) Cellulose content of alcohol‐insoluble cell wall material for *Zmirx15a‐1* and the corresponding WT (*n* = 4). Data represent the mean of at least three biological replicates. *P < 0.05, **P < 0.01; ns, not statistically significant. Student's *t*‐test and Bonferroni correction for multiple tests.

To determine how *ZmIRX15A* regulates SD, we obtained three independent Mutator insertional mutants (UFMu‐08143; UFMu‐08662 and UFMu‐10 974) from the UniformMu mutant library (www.MaizeGDB.org; Figure 4a). PCR analysis coupled with sanger sequencing revealed that the Mutator transposon inserts into the first exon of *ZmIRX15A* in all three mutants, suggesting that all three mutants are likely null (Figure S4A). Under normal growth conditions, none of three mutants exhibited any obvious phenotypes at the seedling stage in comparison with wild‐type (WT; Figure S4B). Phenotypic analysis of the abaxial epidermis in the three mutant lines (*Zmirx15a*‐1/2/3) revealed a significant increase in SD, with mean values ranging from 71 to 77 stomata mm^−2^ compared with a mean density of 66 stomata mm^−2^ in WT leaves (Figure 4b,c). Stomatal size was reduced by approximately 13% in mutants than in WT leaves (Figure 4d), suggesting a negative correlation between these two stomatal traits (de Boer *et al*., 2016; Taylor *et al*., 2012). The stomatal index responsively increased by 14% in mutant plants (Figure 4e). To exclude the influence of growth period and ensure the accuracy of the phenotyping, we also measured third‐leaf length for these mutants during phenotypic identification, which revealed no significant differences (Figure S4C). Taken together, these results indicated that *ZmIRX15A* negatively regulates SD.

Previous work reported that two *ZmIRX15A* homologues in *Arabidopsis*, namely *AtIRX15* and *AtIRX15l*, encode proteins containing the DUF579 motif are essential for normal xylan deposition in the secondary cell wall (Brown *et al*., 2011; Jensen *et al*., 2011). To determine whether *ZmIRX15A* has a similar biological function in maize, sugar composition of the non‐cellulosic fraction of the cell wall was analysed by gas chromatography. The most obvious difference was a decrease in xylose level in the *Zmirx15a‐1* mutant (Figure 4f), and is comparable with that observed in other previously described xylan mutants (Jensen *et al*., 2011, 2014; Mortimer *et al*., 2015). The secondary cell walls of plants are complex structures predominantly comprised of cellulose, lignin and hemicellulose. To determine whether *ZmIRX15A* could have more widespread effects on cell wall deposition, we examined the cellulose content and the results showed that no significant difference between *Zmirx15a‐1* mutant and WT (Figure 4h). Together, these results indicated that *ZmIRX15A* modulates SD by regulating xylan levels in secondary cell wall composition.

### *ZmIRX15A* modulates WUE by altering transpiration and stomatal conductance

**Figure 5:**
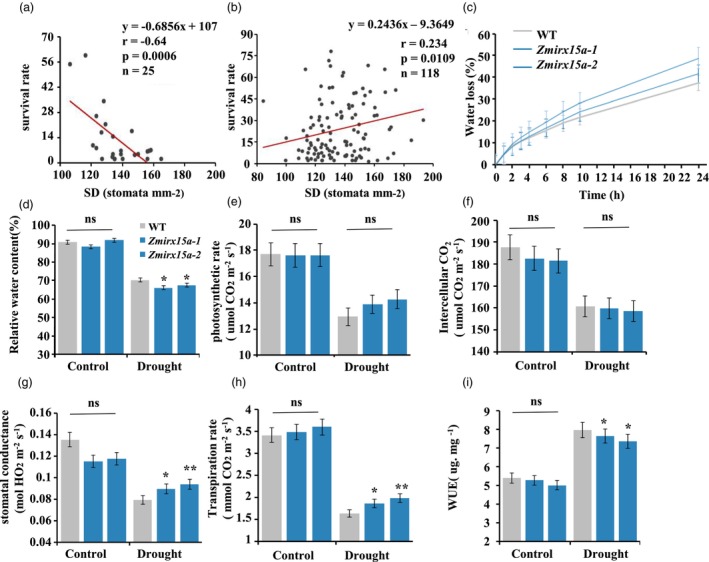
*ZmIRX15A* modulates WUE by altering transpiration and stomatal conductance. (a) Correlation of stomatal density (SD) with survival rate in the SS subpopulation. (b) Correlation of SD with survival rate in the TST subpopulation. (c) Water loss from transpiration in the leaves of wild type and mutants. (d) Leaf relative water content of control and drought‐treated plants after 8 days of treatment. (e–h) photosynthetic rate, intercellular CO_2_, stomatal conductance, and transpiration rate were determined with individual leaves of wild‐type and mutant plants under well‐watered (Control) and drought conditions using a Li‐Cor 6400 gas exchange system. (i) WUE was calculated as photosynthetic rate divided by transpiration rates. Each treatment included three seedlings. Values represent the mean ± SD (*n* = 3). *P < 0.05 or **P < 0.01; ns, not statistically significant. Student's *t*‐test and Bonferroni correction for multiple tests.

SD is closely associated with drought tolerance and WUE in plants (Xiang *et al*., 2021; Yoo *et al*., 2010; Zhang *et al*., 2022). Previous studies were conducted in which survival rate was used as a measure of maize drought tolerance at the seedling stage in the same association population as this study (Mao *et al*., 2015; Wang *et al*., 2016b). To elucidate that whether SD variation contribute to drought tolerance, we attempted to correlate it with drought tolerance via survival rate. The results showed that survival rate correlated negatively with SD in the SS subpopulation (*n* = 25, Figure 5a) but correlated positively with SD in the TST subpopulation (*n* = 118, Figure 5b). To further determine *ZmIRX15A* function in maize under drought stress, *Zmirx15a*‐1 and *Zmirx15a*‐2 together with WT plants were grown to the three‐leaf stage in soil and then subjected to 8 days of water deprivation. The detached leaves of the mutants lost water at a significantly higher rate than WT (Figure 5c). In addition, the relative water content of mutant leaves was significantly less than that of WT leaves (Figure 5d). These results suggested that *ZmIRX15A* negatively regulates the drought tolerance of maize seedlings.

To further examine the physiological mechanisms of *ZmIRX15A* in response to drought stress, we measured photosynthetic rate, intercellular CO_2_, transpiration rate, and stomatal conductance in plants at the three‐leaf stage using a Li‐6400XT portable gas exchange system. Under the water‐sufficient condition, the mutant photosynthetic physiological index mentioned above did not differ significantly from that of WT, whereas all these characteristics were significantly reduced during drought in both mutant and WT plants (Figure 5e–h). In contrast, under the water‐deficient condition, stomatal conductance of mutant was significantly higher than that of WT (Figure 5g). In addition, the photosynthetic rate of mutants increased by 8.8% relative to WT although the difference was not statistically significant, but the transpiration rate increased by 17.4%, resulting in lower WUE (Figure 5e,h). Meanwhile, there is no significant difference in intercellular CO_2_ (Figure 5f). However, WUE (the ratio of photosynthetic rate to transpiration rate) in the mutant plants was significantly lower than that of WT plants (Figure 5i). Taken together, these results suggested that *ZmIRX15A* could modulate WUE by altering transpiration and stomatal conductance without altering the photosynthetic rate or intercellular CO_2_.

### Altered expression of genes related to stomatal complex morphogenesis and drought response

**Figure 6:**
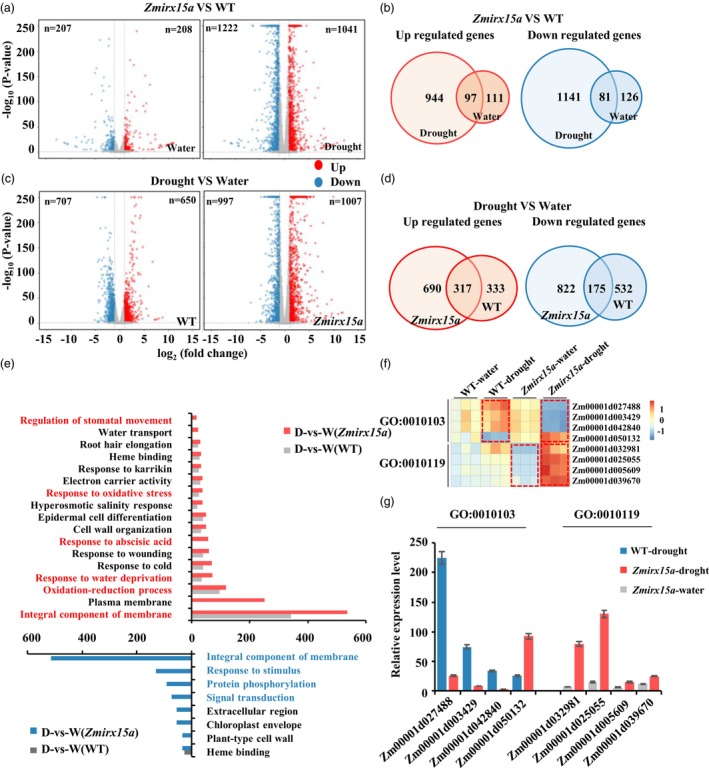
Transcriptome analysis reveals changes in the expression of genes related to stomatal complex morphogenesis and drought resistance. (a) Volcano plots representing the fold change of DEGs in the comparison groups of *Zmirx15a* versus WT under different environmental conditions (*P* < 0.01, absolute fold change ≥2.0). The *x*‐axis and *y*‐axis represent log 2 fold change (FC) and –log 10 (*P*‐value), respectively. The blue and red dots represent down‐regulated DEGs with log 2 (FC) < −1 and up‐regulated DEGs with log 2 (FC) > 1, respectively. The grey dots represent no significant difference in transcriptomes. Three independent experiments were performed for each sample at each time point. (b) Corresponding to the volcano plot on the left, of the DEGs up‐regulated and down‐regulated under different conditions. Volcano plots (c) and Venn diagrams (d) comparing the transcriptomes of drought‐treated with watered conditions with plants having different genotypes. (e) Significantly enriched GO categories associated with the up‐regulated or down‐regulated genes with plants having different genotypes. The *x*‐axis indicates the number of DEGs enriched in a category. (f) Heatmap showing the enriched genes in the GO terms ‘Stomatal complex morphogenesis’ (GO: 0010103) and ‘Regulation of stomatal movement’ (GO: 0010119). (g) qRT‐PCR verification of DEGs shown in (f). D: drought condition; W: watered condition. Data represent the mean ± SE, and significant differences were determined by one‐way analysis of variance.

To understand the molecular processes regulated by *ZmIRX15A*, we compared the transcriptomes of leaves from 3‐week‐old WT (W22) and *Zmirx15a‐1* plants using transcriptome sequencing (RNA‐seq) under well‐watered or drought conditions. Principal component analysis revealed that each set of samples formed distinct clusters, and the three replicates for each treatment clustered together (Figure S5A). The threshold for significantly differentially expressed genes (DEGs) was set at a (log2 scale)‐fold change value ≥1 or ≤−1 and an adjusted *P*‐value <0.05. A total of 208 genes were found to be up‐regulated and 207 down‐regulated in the mutant compared with WT under water replete condition (Figure 6a; Table S4). To evaluate the possible effects of drought, we compared the RNA‐seq data for *Zmirx15a‐1* versus WT and identified 2085 DEGs (944 up‐regulated, 1141 down‐regulated) that were significantly affected by drought in the mutant plants (Figure 6a,b; Table S5). A Gene Ontology (GO) enrichment analysis of DEGs was then carried out using the 21 913 genes expressed in the leaves in this study as a background. The DEGs identified under normal watering conditions were enriched for just six GO terms (*P* < 0.01, false discovery rate <0.05; Table S8); under drought conditions, however, a large number of DEGs were enriched in multiple biological processes and molecular functions (*P* < 0.01, false discovery rate <0.05, Table S9). Interestingly, 44 genes (six up‐regulated, 38 down‐regulated) were enriched in the biological process ‘stomatal complex morphogenesis’ (GO: 0010103, Table S14). The expression of several of these genes was tested independently by RT‐qPCR to validate the DEG results (Figure 6f,g). These results indicated that a portion of these DEGs may be directly or indirectly involved in *ZmIRX15A*‐mediated stomatal development and movement under drought stress.

We also assessed transcriptome differences between drought and normal watering conditions in different genotype backgrounds. Under drought stress, 1357 DEGs (650 up‐regulated, 707 down‐regulated) were identified in the WT background, and 2004 DEGs (1007 up‐regulated, 997 down‐regulated) were identified in the *Zmirx15a‐1* mutant background (volcano plots in Figure 6c; Tables S6 and S7). Comparison of these 1357 and 2004 DEGs revealed 1512 non‐overlapping DEGs as *ZmIRX15A*‐dependent drought‐responsive genes, including 690 up‐regulated and 822 down‐regulated genes (Figure 6d). We also performed GO analysis of up‐ or down‐regulated DEGs to explore the biological processes that involve *ZmIRX15A* (Tables S10–S13). Both up‐ and down‐regulated genes were significantly enriched for response to drought stress, including ‘integral component of membrane’ (GO: 0016021), ‘oxidation–reduction process’ (GO: 0055114), ‘response to water deprivation’ (GO: 0009414), ‘response to abscisic acid’ (GO: 0009737), ‘response to oxidative stress’ (GO: 0006979), and ‘regulation of stomatal movement’ (GO: 0010119; Figure 6e). As expected, most of these enriched terms differed substantially in terms of the number of DEGs in the *Zmirx15a‐1* mutant background. Next, we randomly measured the expression of four genes (GO: 0010119) using qRT‐PCR and found that, consistent with the RNA‐seq results, these genes were expressed at significantly different levels (Figure 6f,g). These results confirmed our previous conclusion for *ZmIRX15A* with respect to is involvement in the regulation of drought‐stress tolerance.

## Discussion

Stomata regulate gas and water exchange in the plant epidermis (Chater *et al*., 2017; Harrison *et al*., 2020), and research has shown that plants having low SD have good potential as genetic donors for improving WUE (Clemens *et al*., 2022; Dunn *et al*., 2019; Franks *et al*., 2015; Guo *et al*., 2019; Schuler *et al*., 2018; Westbrook and McAdam, 2020; Xiang *et al*., 2021; Yoo *et al*., 2010). Here, we found that natural maize‐inbred lines have considerable genetic variability with regard to SD at the seedling stage, and identification of such favourable variations might provide a route for improving maize WUE. In addition, we discovered that InDel1089 underlies the natural variations of SD. Specifically, a naturally occurring 3‐bp insertion decreases the translation of *ZmIRX15A* mRNA by affecting its secondary structure; accordingly, genetic disruption of *ZmIRX15A* increased SD in maize and decreased WUE. Therefore, our identification of *ZmIRX15A* provides a gene target for improving maize WUE by marker‐assisted selection.

### SD evolved to balance yield and drought tolerance in maize natural populations

Maize was domesticated from the wild grass progenitor teosinte (*Zea may* spp.) and originated in southwestern Mexico, and ever since has been cultivated and subjected to extensive selection for traits suited to numerous climatic regions including temperate zones (Doebley *et al*., 2006; Ranere *et al*., 2009). A large number of studies have shown that population structure is significantly associated with differences in various morphological characteristics of maize (Castelletti *et al*., 2020; Liu *et al*., 2021; Sun *et al*., 2022b; Wang *et al*., 2016b), but few studies have addressed the evolutionary variation in SD, which ultimately manifests as geographic phenotypic differences. In our study, maize MIXED and TEM germplasm exhibited a higher SD than TST germplasm, implying that the adaption of maize from tropical to temperate regions was probably accompanied by an increase in SD (Figure 1B). Compared with the tropics, temperate regions tend to receive less rainfall, have long daylight hours, and relatively dry air (Hutjes *et al*., 1998). Studies have shown that stomatal development and morphogenesis are affected by various external stimuli/conditions OR signals, including temperature, light intensity, humidity, and soil moisture content (Casson and Gray, 2008; Qi and Torii, 2018; Xiong *et al*., 2021). Thus, in response to various environmental factors and/or stresses, SD in different maize subgroups has undergone differential adaptation, yet the physiological significance of such differences in adaptive response in maize has remained unclear.

A previous study found that the TST subpopulation exhibited the greatest drought tolerance (based on survival rate) among numerous maize germplasms (Wang *et al*., 2016b). This result implies a classic negative correlation between maize drought tolerance and SD. Our present results, however, correlated SD with drought tolerance via survival rate in a natural maize association population, identified that different subpopulations exhibit diametrically opposite correlations. In the low SD group (TST), drought tolerance (indexed by survival rate) correlated positively with SD, which may be due to the fact that a higher SD results in faster induction of photosynthesis owing to a higher initial stomatal conductance, resulting in greater biomass production and fighting for drought (Sakoda *et al*., 2020). Correspondingly, in the high SD group (SS), the photosynthetic rate may have approached saturation, and drought tolerance (based on survival rate) correlated negatively with SD owing to greater water loss rate resulting from the increase in SD. Therefore, we propose that SD evolved to balance yield and drought tolerance in maize natural populations. Based on these results, exploring the molecular mechanism underlying SD differences caused by such geographic conditions would be of great importance for raising the yield of maize and guiding drought‐resistance breeding.

### Cell wall‐related genes play an important role in morphogenesis of stomata

As a type of glycosyltransferase, IRX15 catalyses the 4‐O‐methylation of the glucuronic acid substituent and plays a role in xylan biosynthesis in the plant cell wall (Brown *et al*., 2011; Urbanowicz *et al*., 2012). Considerable effort has been put forth to identify the glycosyltransferases involved in glucuronoxylan biosynthesis in *Arabidopsis* (Urbanowicz *et al*., 2012; Wu *et al*., 2009, 2010). Our data indicate that the proportion of xylose, a key component of xylan, was significantly reduced in the *Zmirx15a* mutant compared with WT. In addition, *AtIRX15* and *AtIRX15l*, which are homologues of *ZmIRX15A* in *Arabidopsis* have been defined as a new class of genes involved in xylan biosynthesis (Brown *et al*., 2011; Jensen *et al*., 2011). Guard cells, which are distinct from their adjacent epidermal cells, can alter their size and shape reversibly owing to the elastic properties of their cell wall (Jones *et al*., 2003). Recent studies have shown that PECTATE LYASE LIKE12 resides in the guard cell wall to coordinate turgor pressure and wall mechanics for proper stomatal function in *Arabidopsis* (Chen *et al*., 2021). Moreover, single‐nucleus transcriptome results for maize identified a large number of wall‐related genes that may play roles in generating dumbbell‐shaped morphogenesis of developing and mature stomata (Sun *et al*., 2022a). Thus, we suspect that *ZmIRX15A* might regulate and/or coordinate the dynamic cell wall changes that occur between epidermal cells and guard cells during leaf and stomatal development, contributing to SD.

## Materials and methods

### Genome‐wide association study and analysis of expression quantitative trait loci

Stomatal density was determined in an association‐mapping panel composed of 424 diverse inbred lines, which are publicly available at MaizeGo (Li *et al*., 2013; Yang *et al*., 2011). All maize (*Zea mays* L.) inbred lines were planted in pots (30 cm × 20 cm × 17 cm, length × width × depth), then grown in a greenhouse at 22–28 °C under a photoperiod of 14/10 h day/night with photosynthetic active radiation of 200 μmol m^−2^ s^−1^, and were watered daily. The third leaf (3/4 position) from maize at the three‐leaf stage (10 days after seed germination) was phenotyped. To reduce random errors, the SD of the panel was phenotyped in three independent repeats, each of which contained six replicated assays. The GWAS was performed using TASSEL v.3.0 with a mixed linear model, in which the population structure and kinship were estimated as previously described (Bradbury *et al*., 2007; Yu *et al*., 2006; Zhang *et al*., 2010). Approximately 560 000 genomic SNPs with minor allele frequency ≥0.05 were used for the GWAS. The suggestive *P*‐value threshold to control the type I error rate was 1.0 × 10^−5^ (1/85 806), and independent marker numbers were determined by PLINK (window size 50, step size 50, *r* ^2^ ≥ 0.2; Kang *et al*., 2008; Li *et al*., 2012; Purcell *et al*., 2007). The chromosomal positions of the SNPs refer to the B73 reference genome (B73 RefGen_v2). Phenotypic variation explained by a SNP was estimated using the lm function in the R package (https://github.com/fudiyi/code‐for‐publication). TASSEL (version 5.0) was used to analyse expression quantitative trait loci for *ZmIRX15A*, the expression of which (in terms of fragments per kilobase of transcript per million mapped reads) was computed for GWAS analysis using a linear mixed model by incorporating kinship coefficients and population structure.

### Drought treatment and measurement of water loss

Mutator insertion mutants and WT plants (W22 inbred lines) were grown to the three‐leaf stage in the greenhouse under the same conditions as described above. Firstly, the saturated RSWC (Relative soil water content) was approximately 40%, as measured by SU‐LA (Mengchuangweiyie Technology Co., Ltd). Subsequently, water was withheld for 8 days and the RSWC reached ∼8.3% (at this time point, all seedlings were subjected to drought stress). Another group of the same plants was watered daily as a control (Wang *et al*., 2016b). The relative water content of leaves under different treatments was determined as described by Liu *et al*. (2018). In brief, leaves were removed and immediately weighed to obtain leaf fresh weight, then placed into vials filled with distilled water for 24 h to obtain leaf turgid weight. Some leaves were dried to constant weight at 65 °C and reweighed to obtain leaf dry weight. Leaf relative water content was calculated as follows: (fresh weight − dry weight)/(leaf turgid weight − dry weight) × 100%. To determine water loss rate under normal watering, the second leaves were detached at the three‐leaf stage, and their weights were recorded every hour for 12 h at room temperature. The percentage water loss was calculated as (initial fresh weight − final fresh weight)/initial fresh weight × 100% (Liu *et al*., 2018).

### Stomatal density and stomatal length

The lower (abaxial) epidermis of the third leaf (3/4 position) from maize at the three‐leaf stage (10 days after seed germination) was carefully removed using white nail polish and Scotch tape and placed on a glass slide (Yoo *et al*., 2010; Zhang *et al*., 2022). The structure of guard cells and phenotypic characterization of corresponding stomata were clearly observed under a Ci‐S‐FL microscope (Nikon, Tokyo, Japan). Images were acquired with a DS‐Qi2 Microscope Camera system (Nikon). SD (stoma number per area) and stomatal index (ratio of stomata to total epidermal cells, including stomata, stomatal precursor cells, and pavement cells) were calculated based on a leaf area of 1.86 mm^−2^. Stomatal length (guard cell pair length) was measured instead of total guard cell area because of the relatively dynamic nature of stomatal movement. When stomata open or close, the short axis (ventral and dorsal lengths) of the guard cells can increase or decrease but the long axis remains the same. There were three biological replicates for each process.

### *ZmIRX15A* ‐based association analysis

According to the sequences of B73 and W22, eight pairs of primers were designed to amplify the entire *ZmIRX15A* genome (~2.0‐kb fragments) from 275 maize genotypes (Table S15). The sequences were assembled using DNAMAN and aligned with MEGA 5.0. Polymorphisms (SNPs and Indels), which were identified among these genotypes, and their association with the phenotype and pairwise linkage disequilibrium were calculated by Tassel 3.0 under the standard mixed linear model (Bradbury *et al*., 2007; Yu *et al*., 2006; Zhang *et al*., 2010). A *P*‐value of <1.0 × 10^−5^ was used as the final significance cut‐off in the association analysis.

### RNA isolation and qRT‐PCR analysis

WT and *Zmirx15a‐1* plants were cultivated and subjected to drought stress as mentioned above, and another control group was watered daily. Three duplicated cultivations were performed for each group of materials, and five‐cm leaf sections in the middle of the third leaves were harvested from three plants per sample, pooled, and frozen in liquid nitrogen. The samples were stored at −80 °C prior to RNA extraction. Total RNA was isolated from maize leaves or protoplasts using a RNAprep Pure Plant Plus kit (DP441; Tiangen Biotech, Beijing, China), and cDNA was synthesized with the PrimeScript II 1st Strand cDNA Synthesis kit (6210A; Takara, Shiga, Japan). Quantitative real‐time PCR assays were performed with TB Green Premix Ex Taq (RR420A; Takara) using a CFX Connect Real‐Time PCR system (Bio‐Rad). Excepting for the RNA structure experiments, *ZmActin* (Zm00001d034644) was used as an internal control to normalize the qRT‐PCR data. Relative gene expression was calculated using the 2^−ΔΔCt^ method (Livak and Schmittgen, 2001). Table S15 lists the specific primers for qRT‐PCR that were designed according to the relevant sequences.

### RNA secondary structure prediction and RNA structure probing with dimethyl sulphate

The modelling and structure‐prediction programs RegRNA2.0 (http://regrna2.mbc.nctu.edu.tw/) and RNAfold (http://rna.tbi.univie.ac.at/) were used to predict potential two‐dimensional structures of *ZmIRX15A* on account of InDel1089 genotypes and to calculate the MFE in terms of Δ*G*. RNA structure probing with DMS performed as described previously (Caprara, 2013). DMS methylates the N1 position of unpaired adenines and the N3 position of unpaired cytosines, and reactivity to DMS indicates whether the bases are paired. We also carried out control reactions under equivalent conditions in the absence of DMS. To assess any changes in mRNA structure caused by the variant InDel1089, qRT‐PCR experiments were performed using primers DMS‐F1 (nucleotides 188–205 of the *ZmIRX15A* exon2), DMS‐F2 (nucleotides 68–88 of the *ZmIRX15A* 3′UTR), and DMS‐R (complementary to nucleotides 106–125 of the *ZmIRX15A*‐UTR). The truncation rate for InDel1089 mRNA was calculated as follows (Jiang *et al*., 2021):

$$1-\frac{2^{\left(\mathrm{Cq}\left(\mathrm{DMS}-F2/R\right)-\mathrm{Cq}\left(\mathrm{DMS}-F1/R\right)\right)\mathrm{DMS}-\text{treated}}}{2^{\left(\mathrm{Cq}\left(\mathrm{DMS}-F2/R\right)-\mathrm{Cq}\left(\mathrm{DMS}-F1/R\right)\right)\text{untreated}}}$$

(Cq (DMS‐F2/R), PCR cycle number using DMS‐F2/DMS‐R primer; Cq (DMS‐F1/R), PCR cycle number using DMS‐F1/DMS‐R primer).

### Protoplast transient expression assays

To test the effect of variations on gene expression and translation, a dual‐LUC transient expression assay was performed with maize protoplasts. In this system, a REN reporter gene under the control of the 35S promoter was used as an internal control to evaluate protoplast transfection efficiency, and a minimal promoter from the cauliflower mosaic virus (mpCaMV) was inserted upstream of the firefly LUC coding sequence in pGreenII 0800‐LUC, a commercially available dual‐LUC assay vector, to drive the expression of the *LUC* reporter gene. The second exon and 3′UTR of *ZmIRX15A* from lines B73 and BY813 were amplified and cloned into pGreenII0800‐LUC (XbaI) to generate mpCaMV::LUC‐Exon2^B73^, mpCaMV::LUC‐Exon2^BY813^, mpCaMV::LUC‐Exon2 + 3′UTR^B73^, and mpCaMV::LUC‐Exon2 + 3′UTR^BY813^ using the Hieff Clone One‐step PCR Cloning kit (10911ES25; Yeasen Biotechnology). Mesophyll protoplasts were isolated from leaves of 12‐day‐old etiolated B73 seedlings as described (Huang *et al*., 2018). Subsequently, the mesophyll protoplasts were transformed with the prepared plasmids using the previously described polyethylene glycol–mediated transformation method for analysing *LUC* transcript and protein levels (Huang *et al*., 2018). LUC and REN (internal control) activities were assayed using a Dual‐Luciferase Reporter Assay System (Promega) according to the standard protocol. Relative LUC activity was calculated by normalizing LUC to REN activity (LUC/REN). Five biological replicates, each with two technical replicates, were assayed per construct. All experiments were repeated three times.

### Phylogenetic tree construction

The full‐length amino‐acid sequences encoded by *IRX15* genes identified in maize, rice, *Arabidopsis*, and sorghum were obtained from the NCBI (http://www.ncbi.nlm.nih.gov), MaizeGDB (http://www.maizegdb.org) and TAIR (http://www.arabidopsis.org) databases, then aligned using the ClustalX 1.83 program with default parameters. The phylogenetic tree was constructed based on this alignment result using the neighbour‐joining method (Nei, 1987) in MEGA7 (Kumar *et al*., 2016). Evolutionary distances were computed using the Poisson correction method and are in the units of the number of amino‐acid substitutions per site.

### Subcellular localization

To determine the subcellular localization of *ZmIRX15A*, we generated the UBQ10:*ZmIRX15A*‐GFP construct, and a fragment of the *ZmIRX15A* coding region was amplified by PCR from maize cDNA with the primer set *ZmIRX15A*_F_BamH1 and *ZmIRX15A*_R_EcoRI. The amplified fragment was ligated between sites BamH1 and EcoRI of vector pCUN‐NGFP. This construct was introduced into *Agrobacterium tumefaciens* GV3101 and then transferred to tobacco. The abaxial epidermis of transgenic tobacco was analysed with a Zeiss LSM880 confocal microscope using bright‐field and fluorescence imaging. The experiment was performed three times.

### Characterization of the *ZmIRX15A* mutant

Three Mutator transposon mutants (UFMu‐08143; UFMu‐08662; UFMu‐10 974) were obtained from the Maize Genetics Cooperation Stock Center and repeatedly backcrossed into the W22 inbred lines prior to further analysis (McCarty *et al*., 2013). Genomic DNA was extracted, and a PCR‐based assay was conducted to genotype the Mutator insertion at the *ZmIRX15A* locus.

### Physiology measurements

Transpiration rate, intercellular CO_2_, stomatal conductance, and photosynthetic rate were measured with the third leaf of maize plants at the three‐leaf stage using a Li‐Cor 6400 Portable Photosynthesis System, as described by Yoo *et al*. (2010). Conditions in the Li‐6400XT chamber were as follows: constant air flow rate, 500 μmol/s; CO_2_ concentration, 400 μmol/mol; temperature, 25 ± 2 °C; relative humidity, 50%–70%; and photosynthetic photon flux density, 1000 μmol (photon) m^−2^ s^−1^. All measurements were conducted between 09 : 00 and 13 : 00. Water‐use efficiency was defined as the ratio of photosynthetic rate to transpiration rate. The experiment was preformed three times, and three plants were used for each experiment.

### Cell wall analysis and xylan analysis by size‐exclusion chromatography

Non‐cellulosic cell wall sugars were analysed using trimethylsilyl ethers of methyl glycosides, and the cellulose content of stems from 6‐week‐old plants was measured as previously described (Brown *et al*., 2005).

### RNA‐seq analysis

Under drought and normal conditions, the third leaves of five mutant and WT individual plants were collected to conduct the RNA‐seq analysis. The drought treatment and total RNA extraction method were the same as mentioned above. The 150‐bp paired‐end Illumina sequencing was conducted at Berry Genomics (Beijing). An average of 3 Gb of raw data were generated for each sample. Raw sequence reads were trimmed to remove low‐quality bases (*Q* < 20), short sequence reads (length <20), and adapter sequences using Trimmomatic v0.38 (Bolger *et al*., 2014). The trimmed reads were then mapped onto the reference maize genome from MaizeGDB (https://www.maizegdb.org). A total of 21 913 genes were identified, representing 48% of all the predicted genes in maize. The GO analysis was performed using the agriGO classification system (http://bioinfo.cau.edu.cn/agriGO; Du *et al*., 2010; Tian *et al*., 2017). All RNA samples for transcriptome sequencing were also used to validate the mRNA abundance of the DEGs (Figure 6g).

## Conflict of interest

No conflict of interest declared.

## Author contributions

F.Q., Y.H. and Y.Z. conceived and supervised the project. K.Z. and M.X. conducted the experiments. K.Z., Y.H. and Y.Z. prepared the manuscript. All authors read and approved the final manuscript.

## Supporting information


**Figure 1** Structure of *ZmIRX15A* and *AtIRX15* protein.
**Figure 2** Natural variations in maize SD are not associated with *ZmIRX15A* mRNA level.
**Figure 3** Phylogenetic analysis, gene expression pattern, and subcellular localization of *ZmIRX15A*.
**Figure 4** Characterization of the *ZmIRX15A* mutant.
**Figure 5** Summary of the RNA‐seq data from this study.

**Table S1** Phenotypic variation of SD in four subpopulations
**Table S2** GWAS identified 7 genes significant associated with SD in maize seedlings
**Table S3** Variations in the ZmIRX15A genomic region and their association with SD
**Table S4** List of the up‐ or down‐regulated genes in Zmirx15a/WT in Water conditions
**Table S5** List of the up‐ or down‐regulated genes in Zmirx15a/WT in Drought conditions
**Table S6** List of the up‐ or down‐regulated genes in Drought/Water conditions under WT genotype
**Table S7** List of the up‐ or down‐regulated genes in Drought/Water conditions under Zmirx15a genotype
**Table S8** GO analysis of DEGs (*n* = 415) between in Zmirx15a and WT in Water conditions
**Table S9** GO analysis of DEGs (*n* = 2263) between in Zmirx15a and WT in Drought conditions
**Table S10** GO analysis of up regulated DEGs (*n* = 650) between in Drought and Water conditions under WT genotype
**Table S11** GO analysis of down regulated DEGs (*n* = 707) between in Drought and Water conditions under WT genotype
**Table S12** GO analysis of up regulated DEGs (*n* = 1007) between in Drought and Water conditions under Zmirx15a genotype
**Table S13** GO analysis of down regulated DEGs (*n* = 997) between in Drought and Water conditions under Zmirx15a genotype
**Table S14** Gene enriched in the stomatal complex morphogenesis (GO: 0010103)
**Table S15** Primer sequences used in this study
