# Supplementary material for: Natural polymorphisms in ZmIRX15A affect water‐use efficiency by modulating stomatal density in maize
Source: Plant Biotechnol J. 2023 Aug 12;21(12):2560–73. doi: 10.1111/pbi.14153 (PMC10651153; doi:10.1111/pbi.14153)
Supplement: Supplementary file 1 — Figure 1 Structure of ZmIRX15A and AtIRX15 protein. Figure 2 Natural variations in maize SD are not associated with ZmIRX15A mRNA level. Figure 3 Phylogenetic analysis, gene expression pattern, and subcellular localization of ZmIRX15A. Figure 4 Characterization of the ZmIRX15A mutant. Figure 5 Summary of the RNA‐seq data from this study. [file PBI-21-2560-s001.pdf]

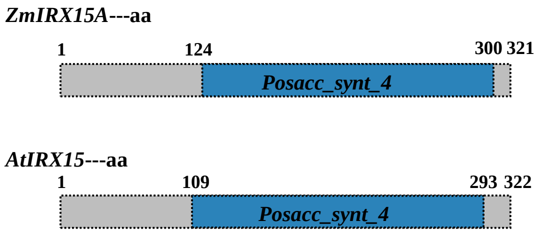

Fig. S1 Structure of *ZmIRX15A* and *AtIRX15* protein

Schematic diagram of the proteins encoded by *ZmIRX15A* and *AtIRX15*. The *Posacc_synt_4* domains are indicated.

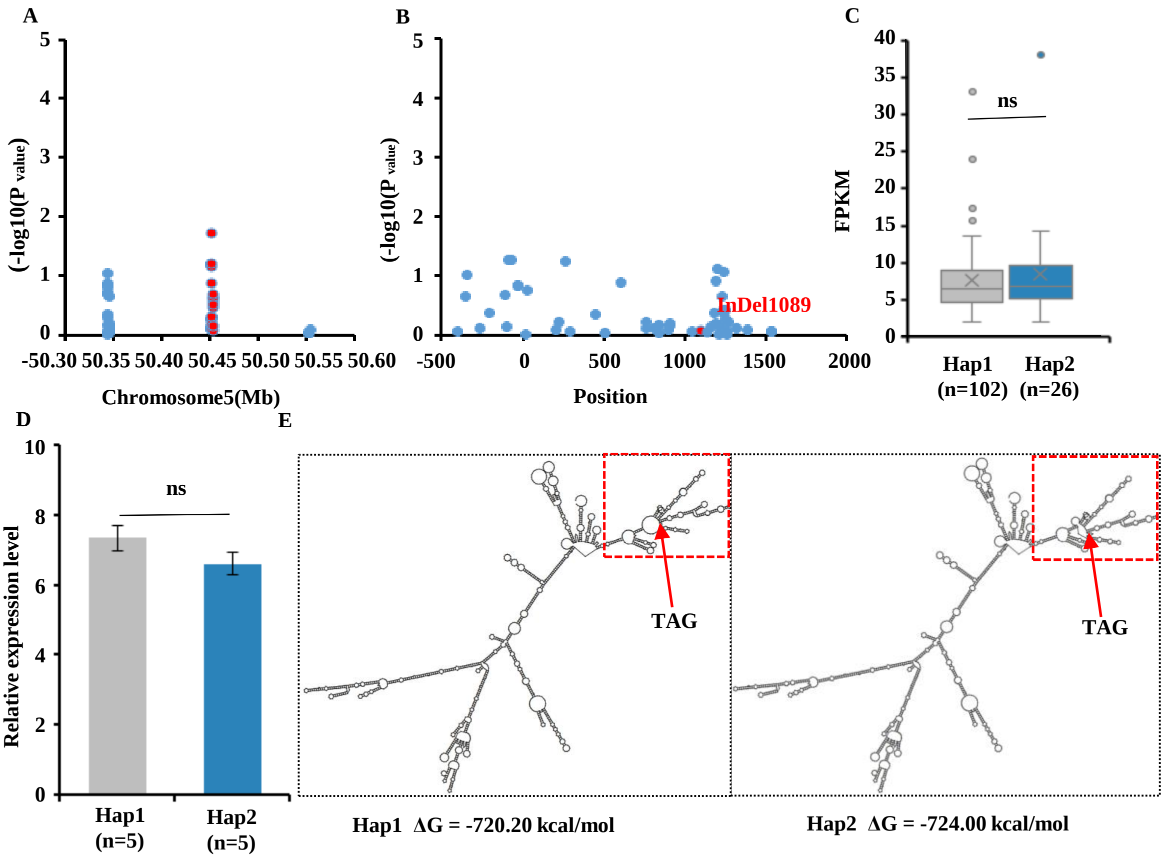

Fig. S2 Natural variations in maize SD are not associated with *ZmIRX15A* mRNA level

A, GWAS revealed that SNPs located in the *ZmIRX15A* region are not significantly associated with *IRX15A* mRNA level. *ZmIRX15A* is indicated in red. A 300-kb region of chromosome 5 is displayed. The association of each marker with *ZmIRX15A* expression was calculated using Tassel software under the standard mixed linear model (MLM, minor allele frequency ≥ 0.05).

B, Association analysis of genetic variation in *ZmIRX15A* with *ZmIRX15A* mRNA level. The most significant variations InDel1089 in the genome-wide association study for SD is highlighted with red.

C, Comparison of *ZmIRX15A* mRNA level calculated using RNA-seq data for the two haplotypes (Hap). ns, not statistically significant. FPKM, fragments per kilobase of transcript per million mapped reads.

D, Transcript levels of *IRX15A* between the two haplotypes as assessed by qRT-PCR. Three replicates for each line were used. n denotes the number of lines.

E, Schematic view of the minimum free energy model of the mRNA secondary structures. The red box indicates the mRNA secondary structural changes caused by InDel1089. TAG: stop codon.

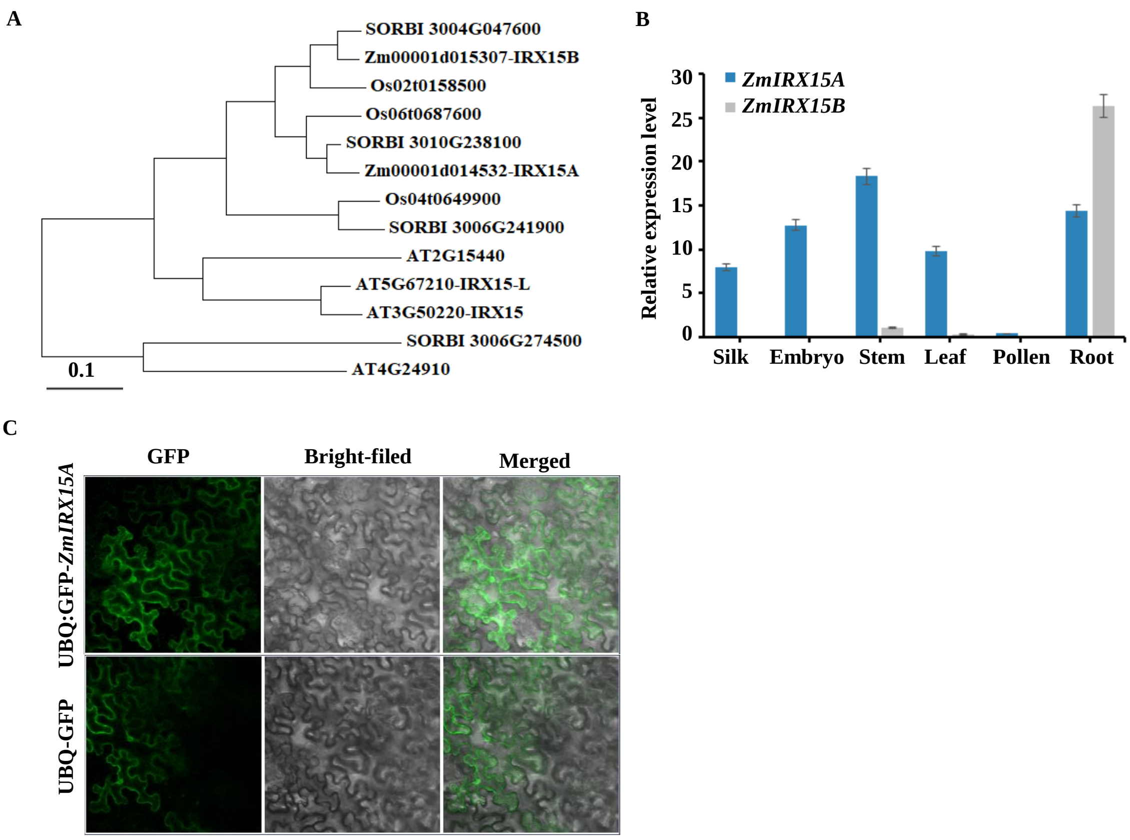

Fig. S3 Phylogenetic analysis, gene expression pattern, and subcellular localization of *ZmIRX15A*

A, Phylogenetic analysis of *ZmIRX15A* and other *IRX* proteins. The analysis involved 11 amino-acid sequences. All positions containing gaps and missing data were eliminated.

B, Expression of *ZmIRX15A/B* in different tissues. Root, stem, and leaf samples were taken from plants at the three-leaf stage, whilst silk and pollen samples were taken from plants at the flowering stage. The gene *Actin* was used as the internal reference.

C, Subcellular localization of GFP-*ZmIRX15A* fusion proteins in UBQ:GFP-*ZmIRX15A* transgenic plants. The left panel shows an image of GFP fluorescence, the middle panel is a bright-field image, and the right panel is a merged image.

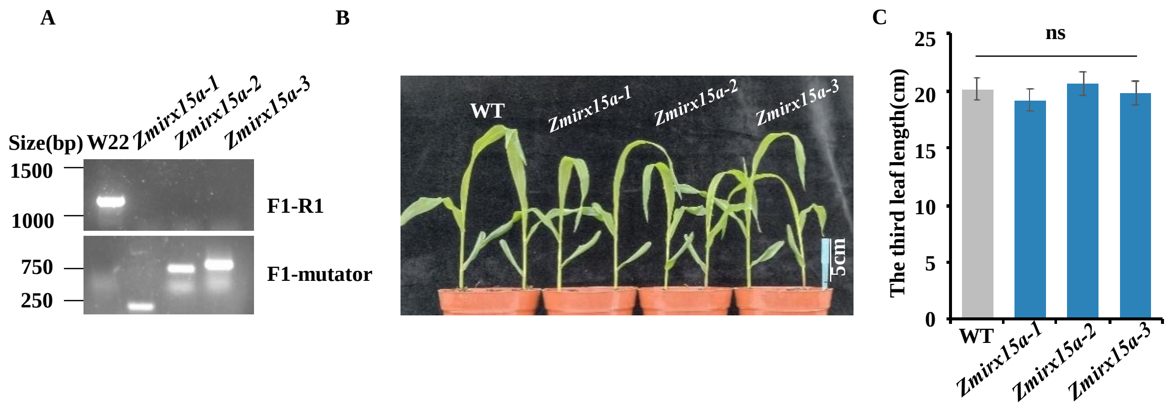

Fig. S4 Characterization of the *ZmIRX15A* mutant

A, Identification of the *Zmirx15a* Mutator-insertion lines by PCR.

B, Morphological comparison of wild-type (left) and *Zmirx15a* mutant (right) seedlings. White scale bar = 5 cm.

C, Ten days after seed germination, no significant difference was found between the third-leaf length of the mutant and the wild-type. Error bars indicate the standard error of the mean. Values represent the mean ± SD (n = 3). ns, not statistically significant. Bonferroni correction for multiple tests.

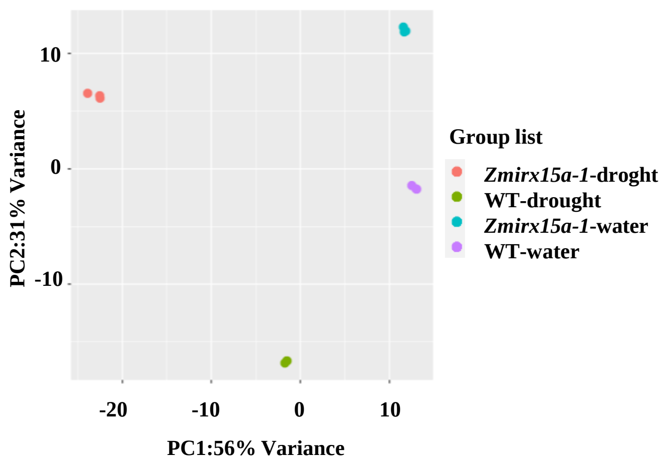

Figure S5. Summary of the RNA-seq data from this study
Principal component analysis plot for all samples.
